# Success rates of pre-hospital difficult airway management: a quality control study evaluating an in-hospital training program

**DOI:** 10.1186/s12245-018-0178-7

**Published:** 2018-03-16

**Authors:** Helmut Trimmel, Christoph Beywinkler, Sonja Hornung, Janett Kreutziger, Wolfgang G. Voelckel

**Affiliations:** 1 Department of Anaesthesiology, Emergency and Critical Care Medicine and Karl Landsteiner Institute of Emergency Medicine, General Hospital Wiener Neustadt, Corvinusring 3-5, A 2700 Wiener Neustadt, Austria; 2 Medical University, Vienna, Austria; 3 Department of Anaesthesiology and Critical Care Medicine, Medical University, Innsbruck, Austria; 4 University of Stavanger, Stavanger, Norway; 5 Paracelsus Medical University, Salzburg, Austria; 6 Department of Anaesthesiology and Critical Care Medicine, AUVA Trauma Center Salzburg, Salzburg, Austria

**Keywords:** Airway management, Prehospital care, Emergency physician, Austria, Difficult airway algorithm, Tracheal intubation, Supraglottic airway, Bag-mask-valve ventilation, Crico-thyrotomy

## Abstract

**Background:**

Competence in emergency airway management is key in order to improve patient safety and outcome. The scope of compulsory training for emergency physicians or paramedics is quite limited, especially in Austria. The purpose of this study was to review the difficult airway management performance of an emergency medical service (EMS) in a region that has implemented a more thorough training program than current regulations require, comprising 3 months of initial training and supervised emergency practice and 3 days/month of on-going in-hospital training as previously reported.

**Methods:**

This is a subgroup analysis of pre-hospital airway interventions performed by non-anesthesiologist EMS physicians between 2006 and 2016. The dataset is part of a retrospective quality control study performed in the ground EMS system of Wiener Neustadt, Austria. Difficult airway missions recorded in the electronic database were matched with the hospital information system and analyzed.

**Results:**

Nine hundred thirty-three of 23060 ground EMS patients (4%) required an airway intervention. In 48 cases, transient bag-mask-valve ventilation was sufficient, and 5 patients needed repositioning of a pre-existing tracheostomy cannula. Eight hundred thirty-six of 877 patients (95.3%) were successfully intubated within two attempts; in 3 patients, a supraglottic airway device was employed first line. Management of 41 patients with failed tracheal intubation comprised laryngeal tubes (*n* = 21), intubating laryngeal mask (*n* = 11), ongoing bag-mask-valve ventilation (*n* = 8), and crico-thyrotomy (*n* = 1). There was no cannot intubate/cannot ventilate situation. Blood gas analysis at admission revealed hypoxemia in 2 and/or hypercapnia in 11 cases.

**Conclusion:**

During the 11-year study period, difficult airways were encountered in 5% but sufficiently managed in all patients. Thus, the training regime presented might be a feasible and beneficial model for training of non-anesthesiologist emergency physicians as well as paramedics.

## Background

In pre-hospital emergency care, airway management is a time-critical intervention of utmost importance. Recent findings underline the competence of the rescuer as key factor in patient outcome, so that formal and on-going training of emergency medical service (EMS) personnel is crucial [1–4]. Compared to the in-hospital setting, the pre-hospital environment is much more challenging, with an up to tenfold higher prevalence of difficult airways [5–7]. For these reasons, training of emergency personnel should have a strong focus on airway management and emergency anesthesia. In contrast to Germany and Switzerland, where the statutory requirements for training of emergency physicians include at least 24 months of postgraduate training, of which 6–12 months are spent in anesthesia and/or intensive care medicine and on-the-job training with mobile emergency services [8, 9], the Austrian curriculum for training of emergency physicians does not include any mandatory clinical practice at all [10]. After doctors have achieved their license to practice as a general practitioner or as specialists of any discipline, they only need to take a 60-h course to qualify as emergency physicians. Under these conditions, it is not surprising that dangerous incidents occur with some regularity in the treatment of emergency patients. Current law in Austria does not require the integration of basic anesthesia training into the education of EMS physicians, although it is undisputed that emergency anesthesia can only be taught properly in clinical practice. In a qualitative control study, we recently demonstrated that non-anesthesiologist EMS physicians are able to achieve a 95% tracheal intubation (TI) success rate when they have had in-hospital training for at least 3 months [4]. Although no fatalities or major complications were observed in the aforementioned analysis, the quality of difficult airway management provided merits further evaluation.

Thus, the purpose of this subgroup and extended data analysis was to review the difficult airway management performance of non-anesthesiologist EMS physicians who underwent a structured airway management training and supervised emergency practice for 3 months, and had on-going training in an anesthesiology department for 3 days per month.

## Methods

**Fig. 1:**
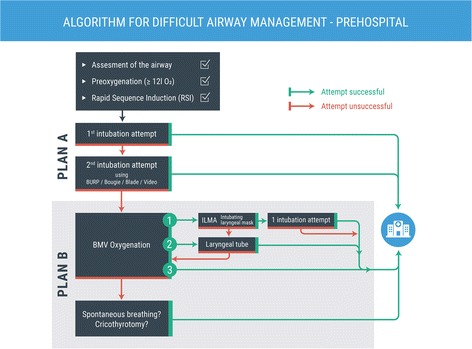
Prehospital Airway Algorithm, Wiener Neustadt Emergency Medical Service

**Fig. 2:**
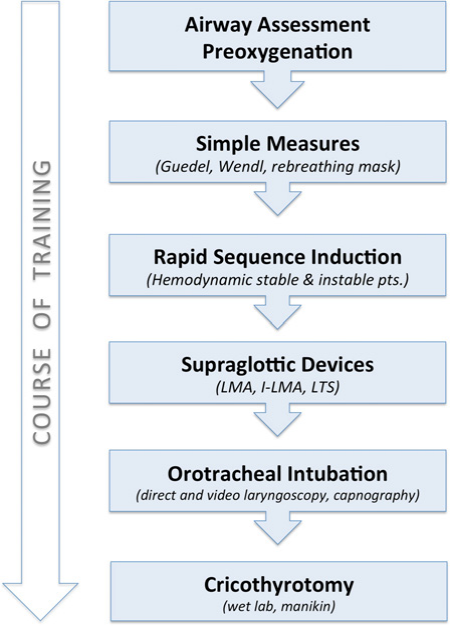
Course of Training for non-anaesthesiologist EMS physicians

At the Landesklinikum Wiener Neustadt, a university teaching hospital in Lower Austria, the department of anesthesiology is responsible for emergency medical service (EMS) for the surrounding region (population approx. 110,000). The majority of the EMS physicians are general practitioners. In the year 2000, a training curriculum for these non-anesthesiologist doctors was established which included expanded theoretical content, simulation training, a 3-month phase of clinical anesthesia practice, and supervised training in mobile emergency service—far beyond the rather rudimentary minimum statutory requirements in Austria [10]. The trainees are taught to assess the airway of anesthetized patients according to a modified “LEMON” method [11], implement the institutional prehospital algorithm for difficult airway (Fig. 1) in the operating room (OR), and learn the surgical airway procedure in wet lab training using the Frova Crico Trainer™ (Habel Medizintechnik, 1210 Vienna) as depicted in Fig. 2. For the duration of their employment as EMS physicians, they are obliged to spend three 6-hour shifts per month in the OR to maintain their skill level in these procedures [4].

To assess the effectiveness of this strategy in everyday pre-hospital practice, we reassessed the data presented in a previous paper [4] and performed this subgroup analysis of pre-hospital difficult airway situations in all emergency physician missions from 1 January 2006 to 31 December 2016, with the approval of the ethics committee for Lower Austria (GS1-EK-4/448-2016V). It is noteworthy that our institutional difficult airway algorithm was established in 2005 and matches with current recommendations such as the European Resuscitation Guidelines. The analysis used data from the electronic EMS recording system “NACA-X™” (EDV Trimmel, 2630 Ternitz) and the hospital information system (HIS; CGM-CompuGroupMedical Austria, 4400 Steyr). Data were processed using “MS Excel™” (Microsoft 98000, Redmond, WA 98052-6399 USA).

## Results

In the period 2006–2016, the emergency physicians of the department were called out 23,060 times; 7352 (31.9%) of these cases were in the NACA^1^ categories 4–7 [12]. The emergency physicians carried out at least temporary ventilation in 933 cases (4.0% of the total or 12.7% of the NACA 4–7 cases). Forty-eight patients (5.1%) were ventilated temporarily with bag mask valve (BMV) only, most often as a supporting measure while waiting for drugs to take effect or during preparation of non-invasive ventilatory support (Table 1). Five patients who had a pre-existing tracheotomy needed emergency intervention because of an acute obstruction or dislocation of the cannula. In 877 patients (94%), TI was indicated, which was successful and confirmed by ETCO_2_ monitoring in 836 cases (95.3%) on the first or second attempt. 64.3% of these patients were in cardiopulmonary resuscitation (CPR) condition while 35.7% needed anesthesia for TI.

**Table 1:** Intermittent BMV—indications

	Number	Mean Age	NACA
Intoxication (sedatives)	4	48.0	5
Intoxication (opioids)	7	24.0	4
Respiratory depression due to analgosedation	3	66.5	5
TI abandonment (do-not-escalate decision)	16	87.0	5
Bridging to NIV	10	68.5	5
Temporary respiratory insufficiency due to seizures	5	1.0	5
Conscious after ROSC	3	57.0	6
Total	48	60.5	5

*NIV* non-invasive ventilation (CPAP via modified Venturi valve and mask), *CPAP* continuous positive airway pressure, *ROSC* return of spontaneous circulation

Forty-four patients (5%) presented with difficult airway: in three cases (0.3%), the airway was secured primarily by means of a supraglottic airway (SGA). In 41 patients (4.7%), attempts to set up TI failed: in 58.9% of these, it was not possible to expose the glottis opening (Cormack and Lehane grade 3 or 4); in 39% of the patients, the view in the airway was too heavily obstructed by vomit or blood. In some cases, the reason for failed TI was given as conditions after operations on the airway (7.3%) or inadequate mouth opening (4.8%). Two patients had to be treated in an extremely restricted space at the scene of the emergency, and in one patient, venous access failed. After failed TI, 22 patients (53.7%) were intubated with a laryngeal tube (LT) in accordance with the institutional DA algorithm (for difficult BMV), and 95.5% of these were successfully ventilated by this method; one patient was switched to intubating laryngeal mask airway (I-LMA). Eleven patients with unproblematic BMV were given an I-LMA after two failed TI attempts. 41.5% of the patients with difficult airway required induction of anesthesia, and 58.5% were under CPR. This was not statistically different to the total sample (*p* = 0.431, data shown above).

Four of the 11 patients ventilated by I-LMA were successfully intubated through the device; seven were brought to hospital with continued ventilation via I-LMA after one intubation attempt. One patient had to be given a crico-thyrotomy because of insufficient SGA ventilation. Eight patients were hospitalized following unsuccessful TI attempts using BMV without any further measures; all but one of these had short distance transport to hospital. In summary, the rate of incidence of difficult airway was 4.6%.

Of the 44 patients with difficult airway, 29 (65.9%) were admitted to hospital. For these, the blood gas results on admission were available in the HIS from 2009 onwards (*n* = 20, 68.9%). These values showed good to adequate oxygenation (p_a_O_2_ > 60 mmHg) in the majority of cases (*n* = 17, 89.4%) but also a high frequency of hypercapnia, both significant (p_a_CO_2_ 50–67 mmHg, *n* = 5) and pronounced (p_a_CO_2_ > 70 mmHg, *n* = 6). Only eight patients (42.1%) were ventilated adequately (Table 2). In the emergency department, 23 (79.3%) of the patients that had had preclinical difficult airway were intubated. Both direct (DL, *n* = 8) and video laryngoscopy (VL, *n* = 3) as well as bronchoscopes (*n* = 4) were used to enable TI; DL was supported by bronchoscopy or tube exchanger (TE) in one case, respectively. In two cases, the LT was changed to TI by using a bronchoscope and a TE. Eleven case records indicated the need for multiple intubation attempts. Intubation was not attempted in two cases due to adequate ventilation by non-invasive methods and in four cases because CPR was discontinued on admission.

**Table 2:** Blood gas analysis at hospital admission

# pat	Airway	p_a_O_2_	p_a_CO_2_	pH	BE	BMI	Outcome
6	LT	117	65	6.92	− 22.6	34.6	D
8	LM	94	66.5	6.9	− 15.7	32.8	D
9	LT	162	81.4	7.09	− 8.8	29.38	D
10	LT	172	64.6	7.18	− 6	24.91	S
11	LT	81	46	7.33	− 1.9	19.57	S
12	LM	261	59	7.24	− 1.3	25.71	D
13	LT	101	55.5	7.21	− 6.3	29.38	S
14	LT	126	36	7.33	− 6	24.48	S
16	LT	405	49	7.31	− 1.8	21.6	S
20	LT	221	43	7.26	− 7.5	29.38	D
21	LT	60	112	7.05	− 0.5	31.56	S
22	LT	67	49	7.19	− 8.7	n.a.	S
23	BMV	188	46	7.3	− 3.1	19.59	S
24	BMV	34	104	n.a.	n.a.	27.75	S
27	LT	25	116	6.81	− 15.3	n.a.	S
31	LM	237	35	7.19	− 14.7	22.22	D
33	BMV	n.a.	n.a.	7.05	− 4.7	n.a.	D
34	BMV	112	74	6.9	− 17	27.76	D
36	LM	146	42	7.37	− 1	29.74	D
40	LT	371	78	7.08	− 6.9	30.25	S
	MV	156.84	64.32	7.15	− 8.06	27.10	
	SD	104.74	24.67	0.17	6.49	4.49	

*pat* patient, *p*_*a*_*O*_*2*_/*p*_*a*_*CO*_*2*_ arterial partial pressure of oxygen/carbon dioxide, *pH* potentia hydrogenii, *BE* base excess, *BMI* body mass index, *S* survived, *D* deceased, *MV* mean value, *SD* standard deviation

In a sample of 47 cardiac arrest patients who already had a laryngeal tube inserted by paramedics during CPR attempts before the emergency physician arrived at the scene, we observed an interesting incidental finding. In 30 patients attended between 2006 and 2013, the LT was left in place throughout CPR. Based on the change resuscitation guidelines, the LT was replaced with TI in 17 patients in the time span between 2014 and 2016. While only one (3.3%) of the LT patients achieved ROSC, five (29.4%) of the TI patients did. This finding is significant (*p* = 0.018), although absolute numbers are small.

On average, over the entire study period, the emergency physicians performed an airway intervention once every 4.3 days or once every 24.7 missions. For an individual emergency physician, this means 10 prehospital TI and 0.4 SGA per year; in the clinical training program, in contrast, they performed 33.5 TI and 19.0 SGA in the first 3 months and 64.0 TI and 33.5 SGA per year during on-going OR training [4].

## Discussion

In this retrospective subgroup analysis, we sought to assess whether a comprehensive in-hospital training program might enable non-anesthesiologist EMS physicians to manage even difficult airway patients. During the 11-year study period, no cannot intubate/cannot ventilate situation was recorded and no patient was harmed due to airway management complications. Sufficient oxygenation was achieved in 90% of the difficult airway patients managed with alternative devices, but hypercapnia was observed in 55% as revealed by admission blood gases (p_a_O_2_ > 60 mmHg and p_a_CO_2_ > 50 mmHg, respectively).

In our opinion, there is a dilemma in emergency medicine: the influence of TI on the outcome of emergency patients may reflect the different qualification levels of EMS personnel, and the main reason for difficulties is lack of practice. Pre-hospital TI is an extremely rare experience for an individual emergency physician or paramedic. In an analysis of over 82,000 emergency physician missions, Gries et al. found an intubation frequency of 1 per 0.5–1.5 emergency physician months [13, 14]; in our EMS system, the frequency of invasive airway interventions was similarly low (4% of all missions). Therefore, a sufficient level of skill can neither be acquired nor maintained in EMS alone. To achieve a 90% success rate of TI with direct laryngoscopy requires the experience of performing 50–150 procedures [15, 16]. Besides learning the manual skills, constant training and feedback from experienced practitioners is needed [17, 18]. By our training program, emergency physicians were enabled to perform approximately 75 TIs per year and receive feedback and supervision in the operation room [4].

Clinical competence and self-efficacy is of particular importance when airway management becomes difficult. In emergency medicine, the airway must often be secured under considerable time pressure, usually with the patient lying on the ground and unable to cooperate. Commonly used predictors for difficult intubation from the clinical setting are often less useful [19]. Murphy and Walls recommend the “LEMON” method for emergency medicine: comprising the external appearance (*l*ook), easily estimated parameters such as the mouth opening and chin to thyroid distance (*e*valuate), the *M*allampati score, and checks for *o*bstruction and *n*eck mobility [19]. We implemented a modified version, omitting the Mallampati score and trained emergency physicians in the “LEON” method (Table 3) on sedated and anesthetized patients [11]. The number of cases with predicted difficult airway, in which SGA was chosen as the primary method of airway management, was low in our study (*n* = 3). Nevertheless, in 24/41 patients, attempts to set up TI failed mainly because it was not possible to expose the glottis opening (60%) or due to airway obstruction with vomit or blood (39%). Thus, EMS physicians are challenged to handle unexpected, unrecognized difficult airways or, to put this the other way round, they must approach every airway as though it could be difficult.

**Table 3:** Airway assessment by modified “LEON” method [20]

Airway assessment: modified “LEON” method	
Look	Facial trauma Abnormal facial shape (craniofacial deformities) Teeth - protruding - large incisors - false teeth? Large tongue Beard Obesity
Evaluate	Incisor distance > 3 fingers Hyoid/mentum distance (mandible length) > 3 fingers Thyroid to floor of mouth > 2 fingers
Obstruction	Obstructed airway (tissue swelling, foreign body, obesity)
Neck	Neck mobility (if feasible)

Algorithm-based procedures can improve success rates of interventions and patient outcome [20]. Our results confirm for the EMS setting what Berkow et al. [21] demonstrated for clinical anesthesia: structured clinical training and algorithm-based treatment of patients improve patient safety. Of 41 patients whom non-anesthesiologist emergency physicians were unable to intubate, 32 were ventilated successfully using a supraglottic device and 8 via BMV, and crico-thyrotomy was needed in a single case only (0.12%). Furthermore, there were a number of cases where emergency physicians were reluctant to use TI or SGA in favor of less-invasive procedures (*n* = 48, Table 1). This can be interpreted as a rational and appropriate deployment of airway management methods, enabled by thorough training.

Incorrect positioning and wrong handling of an SGA device can also cause relevant complications. For the LT, several problems have been noted, such as impairment of cerebral perfusion due to excessive cuff pressure of up to 100 mmHg during CPR [22]. Thus, we changed our standard operating procedure in 2014 in favor of immediate conversion of all LTs to TI. Interestingly, we noticed a return of spontaneous circulation rate in patients prior to 2014, with on-going LT ventilation of 3.3%, compared to 29.4% when the LT was replaced by TI. However, it is noteworthy that numbers are too small to draw any conclusion from this observation.

Finally, training in the OR covers more topics than just bag-mask ventilation and placement of laryngeal or tracheal tubes. Learning the correct rapid sequence induction technique, ventilation, and hemodynamic management of emergency patients is equally important [23]. Unfortunately, Austrian law on training of emergency physicians [10], which dates from 1998, does not provide anything more detailed than an outline curriculum of some theoretical topics to be taught. As a consequence, rescuer qualifications as well as institutional approaches are likely to vary significantly between EMS systems in Austria.

Some limitations of our retrospective quality control study must be noted. First, this is a retrospective study and we are unable to provide detailed information about time intervals, SpO_2_, and first etCO_2_ levels during and after difficult airway management. Second, we are unable to provide detailed information whether the quality of airway management had an impact on outcome. The goal of our study was to assess whether life-threatening pre-hospital difficult airway situations can be successfully managed even by non-anesthesiologists with proper training. Thus, we can only discuss the feasibility and efficacy of our educational approach for EMS physicians. Accordingly, our study lacks a potential control group.

## Conclusions

Our data indicate that a training period of 3 months, followed by regular training in the OR, is sufficient to impart an adequate level of competence in emergency difficult airway management. Strict adherence to an institutional algorithm enabled catastrophic “cannot intubate/cannot ventilate” situations to be avoided. Thus, we believe compulsory clinical training should be included in legal requirements for both emergency physicians and paramedics.

## Footnotes

1 *N*ational *A*dvisory *C*ommittee of *A*eronautics

## References

[1] Wang HE, Davis DP, Wayne MA, Delbridge T (2004). Prehospital rapid-sequence intubation-what does the evidence show?.

[2] Lecky F, Bryden D, Little R, Tong N, Moulton C, Lecky F (1996). Emergency intubation for acutely ill and injured patients.

[3] Fullerton JN, Roberts KJ, Wyse M (2011). Should non-anaesthetists perform pre-hospital rapid sequence induction? An observational study. Emerg Med J.

[4] Trimmel H, Beywinkler C, Hornung S, Kreutziger J, Voelckel WG (2017). In-hospital airway management training for non-anesthesiologist EMS physicians: a descriptive quality control study. Scand J Trauma Resusc Emerg Med.

[5] Timmermann A, Eich C, Russo SG, Natge U, Bräuer A, Rosenblatt WH (2006). Prehospital airway management: a prospective evaluation of anaesthesia trained emergency physicians. Resuscitation.

[6] Breckwoldt J, Klemstein S, Brunne B, Schnitzer L, Mochmann H-C, Arntz H-R (2011). Difficult prehospital endotracheal intubation—predisposing factors in a physician based EMS. Resuscitation.

[7] Lockey DJ, Avery P, Harris T, Davies GE, Lossius HM (2013). A prospective study of physician pre-hospital anaesthesia in trauma patients: oesophageal intubation, gross airway contamination and the “quick look” airway assessment. BMC Anesthesiol.

[8] Deutschland B. Weiterbildungsordnung Fassung 2015 [Internet]. bundesaerztekammer.de. Available from: http://www.bundesaerztekammer.de/aerzte/aus-weiter-fortbildung/weiterbildung/muster-weiterbildungsordnung/. Accessed 10 Feb 2018.

[9] Fortbildung SIFÄWU. Notarzt SGNOR [Internet]. Available from: https://www.fmh.ch/files/pdf20/fa_notarzt_version_internet_d.pdf. Accessed 10 Feb 2018.

[10] Österreichisches Ärztegesetz [Internet]. Available from: https://rdb.manz.at/document/ris.n.NOR40049274. Accessed 10 Feb 2018.

[11] Soyuncu S, Eken C, Cete Y, Bektas F, Akcimen M (2009). Determination of difficult intubation in the ED. Am J Emerg Med.

[12] Tryba M, Brüggemann H, Echtermeyer V (1980). Klassifizierung von Erkrankungen und Verletzungen in Notarztrettungssystemen. Notfallmedizin.

[13] Gries A, Zink W, Bernhard M, Messelken M, Schlechtriemen T (2006). Realistische Bewertung des Notarztdienstes in Deutschland. Anaesthesist.

[14] Deakin CD, King P, Thompson F (2009). Prehospital advanced airway management by ambulance technicians and paramedics: is clinical practice sufficient to maintain skills?. Emerg Med J.

[15] Konrad C, Schüpfer G, Wietlisbach M, Gerber H (1998). Learning manual skills in anesthesiology: is there a recommended number of cases for anesthetic procedures?. Anesth Analg.

[16] Schüpfer GK, Konrad C, Poelaert JI (2003). Manual skills in anaesthesiology. Anaesthesist.

[17] Kovacs G, Kovacs G, Bullock G, Bullock G, Ackroyd-Stolarz S, Ackroyd-Stolarz S (2000). A randomized controlled trial on the effect of educational interventions in promoting airway management skill maintenance. Ann Emerg Med.

[18] Bernhard M, Mohr S, Weigand MA, Martin E, Walther A (2011). Developing the skill of endotracheal intubation: implication for emergency medicine. Acta Anaesthesiol Scand.

[19] Gaither JB, Stolz U, Ennis J, Moiser J, Sakles JC (2015). Association between difficult airway predictors and failed prehospital endotracheal intubation. Air Med J.

[20] Thies K, Gwinnutt C, Driscoll P, Carneiro A, Gomes E, Araújo R (2007). The European trauma course—from concept to course. Resuscitation.

[21] Berkow LC, Greenberg RS, Kan KH, Colantuoni E, Mark LJ, Flint PW (2009). Need for emergency surgical airway reduced by a comprehensive difficult airway program. Anesth Analg.

[22] Schalk R, Byhahn C, Fausel F, Egner A, Oberndörfer D, Walcher F (2010). Out-of-hospital airway management by paramedics and emergency physicians using laryngeal tubes. Resuscitation.

[23] Miller M, Kruit N, Heidreich C, Ware S, Habig K, Reid C, Burns B (2012). The frequency and significance of postintubation hypotension during emergency airway management. J Crit Care.

